# Pharmacist perceptions on the presentation of social isolation and loneliness (SIL) in community pharmacy settings in Ireland – A mixed methods study

**DOI:** 10.1016/j.rcsop.2025.100686

**Published:** 2025-11-20

**Authors:** Jung-Ah Hong, Hebah Al-Rashed, Laura Rice, Fabian F. Sweeney

**Affiliations:** School of Pharmacy and Biomolecular Sciences, RCSI University of Medicine and Health Sciences, St Stephens Green, Dublin, Ireland

**Keywords:** Loneliness, Social isolation: Community pharmacy, Primary care: Public health

## Abstract

Social isolation and Loneliness (SIL) represent a critical public health issue as they are strongly associated with adverse physical, mental and emotional health and wellbeing outcomes. Community pharmacies are accessible and frequently accessed healthcare locations and are potentially valuable settings for interventions designed to support patients experiencing SIL. However, a barrier to the development of community pharmacy based SIL interventions is a knowledge gap regarding nature of the presentation of SIL in this setting. This study adopts a mixed methods approach to explore this from the perspectives of practicing community pharmacists in Ireland.

Semi-structured interviews were conducted with community pharmacists practicing in Ireland (*n* = 9) followed by a national survey of practicing community pharmacists (*n* = 95 participants).

Four themes were identified from the interview data: **recognition of SIL, SIL risk factors, enablers** and **Barriers to supporting patients experiencing SIL**. Pharmacists described frequent encounters with SIL particularly among older and underrepresented patents. SIL was often recognised through the presence of pharmaceutical care issues and other indirect approaches. Pharmacists expressed a strong sense of responsibility to support patients. Barriers to supporting patients were reported such as lack of appropriate training, signposting knowledge and resource constraints. Survey findings corroborated these themes with respondents reporting frequent encounters with SIL, and needs for guidance and resourcing support.

This study highlights the potential for pharmacies to act as valuable locations for interventions supporting social connectivity. However, integration of pharmacy services into wider support and wellbeing services, appropriate resourcing, inclusion of SIL in pharmacy education, and appropriate patient informed service co-design are critical for this potential to be fully realised.

## 1 Introduction

Social isolation and Loneliness (SIL) are increasingly recognised as pressing public health issues internationally.^1^ Social isolation, is widely conceived of as where individuals have objectively infrequent social contact,^2^ whereas loneliness describes the complex subjective distressful feelings that an individual's level of social contact is inadequate for their needs.^3^ SIL are robustly associated with poorer physical, mental, cognitive and emotional health and mortality outcomes.^2^^,^^4^ In response to this public health issue, pharmacological, cognitive based therapy (CBT) and social prescribing-based approaches to support patients experiencing SIL have been developed and evaluated.^5^, ^6^, ^7^, ^8^ SIL are prevalent internationally, however Ireland reports a specifically high level of loneliness^9^ marking it as a significant national public health priority.^10^

Prior research has considered the role of health professionals, particularly general practitioners,^11^^,^^12^ in supporting patients experiencing SIL, however less consideration has been given to community pharmacies in this context. This represents a notable evidence gap, considering that community pharmacies, as accessible healthcare settings and locations for social interaction with a rich understanding of individual and community healthcare needs, are well-positioned to support patients experiencing SIL. Indeed, moderate loneliness has been suggested as being highly prevalent among individuals accessing community pharmacy services in Ireland.^13^ Ongoing expansion of community pharmacy roles in Ireland to include prescription extension and delivery of common illness prescribing services^14^ further suggests that exploration of the role of the pharmacy in delivering wider health and wellbeing services, including for SIL, is timely.

Internationally, several initiatives have attempted to address SIL in community pharmacy settings, with interventions focusing on screening for social determinants of health, engagement with local support services, and participation in social prescribing networks.^15^, ^16^, ^17^, 18., 19, 20 Several of these interventions have encountered challenges related to successful implementation and scaling,^18^^,^^19^ and involve multiple stakeholders and behaviours. This suggests that appropriate tailoring of these interventions may be required for the specific context of Irish community pharmacy to reflect the high reported rates of loneliness in Ireland compared to other EU states,^9^ specific workflow considerations and specific situation within the Irish health and social care systems.

The nature of how loneliness and social isolation presents in Irish community pharmacy settings has yet to be thoroughly described from a phenomenological perspective. A rich understanding of this will support the adaptation and development of novel and effective SIL interventions and practice support resources acceptable to both pharmacy teams and patients. This research aims to begin addressing this knowledge gap by exploring the experiences and perspectives of practicing community pharmacists in Ireland, regarding the presentation of patients experiencing SIL in the course of their professional practice.

## 2 Methods

### 2.1 Research methodology

To develop a deeper understanding of the presentation of SIL a mixed methods approach was adopted. This consisted of a phenomenological qualitative enquiry based around semi-structured qualitative interviews and inductive thematic analysis, followed by a quantitative survey study for triangulation.

### 2.2 Ethical approval

The study was conducted with ethical approval from the RCSI University of Medicine and Health Sciences research ethics committee (Refs: REC202305020 and REC202406014).

### 2.3 Semi-structured interviews

#### 2.3.1 Recruitment

Community pharmacists practicing in Ireland were recruited using a purposive and convenience sampling approach combined with a snowball recruitment strategy. These approaches were adopted to ensure inclusion of individuals with appropriate experience within a feasible timescale, while also expanding beyond initial networks to broaden perspectives gathered. Community pharmacists currently working in the Republic of Ireland, who were willing and able to participate in an interview using the MS Teams platform were considered eligible for participation. A participant information leaflet, outlining the aims, purpose and risks of the study form were distributed to all participants before data collection. All participants provided written informed consent to participate in this study. Recruitment and thematic analysis were conducted simultaneously with recruitment ceasing once data saturation, that is the point where new themes were not emerging from analysis of interview transcripts, was observed.^21^ A total of nine pharmacists (Table 1) were recruited and interviewed.

**Table 1:** Details of semi-structuctured interview participants.

Participant number	Gender	Experience (Years)	Current Role	Primary Pharmacy Location
1	F	34	Pharmacy owner, supervising and superintendent pharmacist	Dublin suburb
2	M	21	Supervising pharmacist	Dublin suburb
3	F	20	Support pharmacist	Dublin suburb
4	F	7	Locum pharmacist	Dublin city centre
5	M	15	Pharmacy owner, supervising and superintendent pharmacist	Dublin suburb
6	F	2 months	Support pharmacist	Dublin suburb
7	F	20	Pharmacy owner, supervising and superintendent pharmacist	Small town in Munster
8	F	20	Supervising pharmacist and superintendent pharmacist	Large town in Connacht
9	F	16	Supervising pharmacist	Large town in Leinster

#### 2.3.2 Semi-structured interviews

A topic guide was developed iteratively through pilot testing with two practising community pharmacist volunteers. Interviews were conducted with these volunteers, with minor amendments made to the topic guide in terms of wording and topic flow based upon researcher reflection and participant feedback. Transcripts of these pilot interviews were not included in the final inductive analysis.

Interviews were conducted on a one-to-one basis using the Microsoft Teams platform, at a time of the participants convenience. Interview duration was an average of 40 min (range 31 min to 58 min). No financial incentive was provided to participants. Interviews were conducted by FS, an experienced community pharmacist and lecturer in pharmacy practice. The topic guide used is included as supplemental information (S1).

#### 2.3.3 Data analysis

Interviews were recorded and transcribed, and inductive thematic analysis was conducted. A collaborative approach to analysis was employed as described in the literature,^22^^,^^23^ which involved initial open and axial coding of transcripts, development and pilot testing of a codebook, and final thematic analysis of the transcripts. JH, HA (Senior MPharm students completing community pharmacy internships, with initial training in conduction qualitative research provided by FS) conducted the thematic analysis collaboratively with FS facilitating regular discussions to clarify codes, themes and consider how the research teams own backgrounds may have shaped interpretation of data. Points of disagreement between the two primary reviewers were resolved by FS.

### 2.4 Survey study

A quantitative survey was developed based on findings from thematic analysis of semi-structured interviews. Inclusion criteria for the study was registered pharmacists who regularly practice in community pharmacy settings in Ireland. The survey explored participant information and practice setting (3 questions), experiences of presentations of loneliness and social isolation (4 questions), professional and pharmacy team roles in supporting patients experiencing SIL (3 questions), perceived self-efficacy in identifying patients experiencing SIL (1 questions), barriers to supporting patients experiencing SIL (2 questions), experiences with referring patients to community based SIL support services (1 question), perceived enablers to supporting patients with SIL (1 question). Questions consisted of multiple choice and Likert scale questions, and included the ability to provide open ended responses, including overall thoughts. The survey was presented over a single screen. The survey questionnaire is included as supplemental information (S2).

This survey was piloted with practicing community pharmacists and iteratively developed for clarity and brevity. The survey was tested for technical functionality, before distribution via email to a list of practicing community pharmacist(*n* = 6254). This list is maintained by the Pharmaceutical Society of Ireland (PSI), the statutory regulatory body for pharmacists in Ireland. It is comprised of individual pharmacists who have both consented to their email address being used for academic research participation purposes, and who have described their primary field of practice as community pharmacy during the process of their initial and annual re-registration as pharmacists. Participation was voluntary and anonymous, without incentive. Informed consent was sought from participants via the questionnaire form, with non-consenting individuals exited from the survey. The survey was presented and hosted securely using the web-based Microsoft Forms application. Responses were open for one month from the distribution of the survey, which was administer in October and November 2024. Responses to Likert and multiple-choice questions were analysed using descriptive summary statistical methods. 95 % confidence intervals for responses were calculated using Wilson's score. Open-ended responses were reviewed for patterns and for illustrative insights.

## 3 Results

### 3.1 Semi structured interviews

Details of participants, including their practice experience, role and geographical location are outlined in Table 1.

### 3.2 Thematic analysis

Thematic analysis generated four themes with accompanying sub-themes. The four main themes were **recognition of SIL, SIL risk factors, Enablers for supporting patients experiencing SIL** and **Barriers to supporting patients experiencing SIL.** These themes with accompanying subthemes are outlined in Table 2.

**Table 2:** Themes and subthemes.

Themes	Subthemes
Recognition of loneliness and social isolation in community pharmacy	Impact of loneliness and social isolation on health
	Structural and Functional Perception
	Indirect approach
	Pharmaceutical care prompts intervention for isolated patients
Social Isolation Risk Factors	Older patients
	Member of Underrepresented population
	Personal experiences and social issues
Enablers to interventions	Member of the community
	Existing healthcare partnerships
	Experience
	Pharmacy team-patient rapport
	Pharmacy staff as pharmaceutical and social support
Barriers to interventions	Referral pathways awareness
	Pharmacy Workflow Pressures
	Lack of Training

### 3.3 Theme 1 - Recognition of SIL

Participants frequently described encounters with patients experiencing loneliness and social isolation as a salient and recognisable element of their professional practice. Indeed, Participants described experiences where they had encountered patients experiencing often severe health and social care issues, in which they believed SIL were important contributing factors.

*“What I would see from that is certainly their physical health is not being cared for as much because they do not have anyone to encourage them to either go to the doctor more or to look after their health, or even something as simple as eat more.” - P1.*

*“So like from that point of view, she's so isolated. She's so lonely. There's people who know about it. But no one could Kind of go in and help her, do you know. And then it ended it up, went into the river. Suicide.” - P9.*

Loneliness and social isolation were not considered separately by participants, with most participants using descriptions of loneliness more strongly aligned with structural and functional perspectives closer to social isolation and related to quantity and utility of social contacts. Emotional aspects of loneliness were less salient for participants.

*“I understand loneliness to be people who would not have much interaction or would have little support from friends or family or from just external contact outside their own house.” - P5.*

*“People who have been, you know, are kind of less engaged in the Community and maybe people living from living on their own elderly people in particular, I think, be a bit more vulnerable people as well, who may have over the years have less contact with family members through disputes and fallen out and things like that.” – P2.*

*“They would probably just be living by themselves, and they wouldn't have much contact or support with other people, so there would pretty much just be looking after them themselves and sort of quite isolated or whatever and loneliness again. I would probably put that down as living by yourself and not having much contact or visitors or you know, things like that.” – P3.*

Participants described situations where they had intervened in cases where loneliness or social isolation was suspected. Related to consideration of SIL as a predominantly structural and functional issue, these interventions were prompted by emerging pharmaceutical or medical care issues, as opposed to concerns regarding wellbeing issues arising from loneliness.

*“Because when he was not coming in for his medication, I would ring his brother, the younger brother. And why has it not been collected? Like we can see on ground level of what is going on.” - P7.*

*“I noticed she wasn't coming in for a medication. And so I rang the doctor and spoke to the doctor. And it just sort of flagged it with him and say, listen, I'm concerned.” - P2.*

*“The poor lady was an alcoholic and she wasn't, in my view, being compliant [with her medicines] and wasn't answering her phone… I ended up getting in the car and driving to her house.”– P10.*

Considering their own approaches to identifying and engaging with patients potentially experiencing SIL, participants described adopting indirect approaches to raise SIL as an issue with patients.

*“It is not something I would say, like do you suffer from loneliness? You know. Do you suffer from isolation?” - P8.*

*“I probably would you know more, get the information without using either social isolation or loneliness in the conversation. In you know what he up to today and what's your plan for the week as opposed to i.e., I suppose stating it as a problem. That's how I'd normally, but I wouldn't have a problem talking about it at all with my patients.”– P1.*

Participants offered reasoning for this approach based upon a recognition of both the potential sensitivity of SIL as a conversation topic, and an awareness of stigma associated with SIL, including the potential for self-stigma.

*“I would be like you know, how are you? I would generally without saying the words, broach the subject. People do not want to be told they are alone. It identifies to them that they are isolated…and you do not want to tell someone that they have nobody. They do not want to hear it makes them sadder.” – P5.*

### 3.4 Theme 2 - social isolation risk factors

Certain patient populations with an increased risk of experiencing SIL were considered as being particularly relevant in the context of community pharmacy practice.

Older patients were particularly recognised as individuals with converging risk factors for loneliness and social isolation, and regarded as particularly salient as frequent users of pharmacy services with greater pharmaceutical care needs.

*“…not to say that it is not obviously an issue for younger people, but I feel it would be more on our radar because you know the hours we are in contact with the elderly is much greater.” - P2.*

*“like plenty of people that are younger can be lonely and alone. And isolated, but I suppose stereotypically, I would think of an older person. Maybe would old and living on their own and you know, maybe doesn't drive her, only have to drive in once a week or you know, that kind of thing.” – P9.*

*“I just think that we as a profession would see more older people due to the health issues” - P5.*

Some participants also recognised other under-represented groups with converging risk factors for loneliness who frequently utilise pharmacy services. These included people experiencing or recovering from addiction or other mental health issues, people from migrant communities and neuro-divergent individuals.

*“Asylum seekers and they would be quite socially isolated as well. So, they have the added factor of language barrier and so that makes it challenging.” - P3.*

“*…you can't just leave it as older cause like there's neurodiverse people – P5.*

*“some of your methadone patients that are in hostels and things like that. I think it's the addiction that has led to isolation from their family and isolation from like the, you know, sort of the normal supports or whatever. So they're quite and then it's leads to sort of a chaotic lifestyle, which probably leads to more isolation or whatever. And then also because of the housing situation that then find themselves in.” - P3.*

*“We would dispend a bit of methadone here. Like we have a few people, you know, it would have been drug users, former drug users and good to use their cases cause that and be clean for a long time. You know where isolation would be an issue, and again complex needs.” - P2.*

*“Psychiatric patients… who would collect their prescription every week because they can't take them more often because they would obviously be a risk of overdosing and for them we see them every week and they come in and a lot of them would be very much isolated from you know when they're that severely under medication that they have to be dispensed weekly, I think they wouldn't have a huge amount of social outlets, so we would see them. - P1.*

Related to this was a recognition of the many primary causes of loneliness and social isolation including experience of physical or mental illness or housing issues.

*“They're living in a home. They're not short of money, but what they are short of is I suppose a guardian or a, you know, just some sort of a social structure.” - P1.*

*“I suppose in the context of community pharmacy. Often the people that I would perceive to be like, lonely or socially isolated through like their experience or through their illness or like to their experience, might be like caring for another person or through like homelessness, drug connection.” - P4.*

*“People's lifestyles, working from home, like an elderly person, maybe they didn't get married, they don't have a family. Maybe their family lives abroad.” P7.*

### 3.5 Theme 3 - enablers for supporting patients experiencing SIL

Several aspects of the community pharmacy setting, and the presentation of SIL in pharmacies were identified as enablers to supporting patients experiencing SIL.

Participants strongly identified themselves as members of their local community and identified this as a strong motivator for intervening and supporting patients experiencing loneliness or social isolation. Indeed, participants often described concern for patients experiencing SIL as aligning with their local community membership, as much as their professional responsibility.

*“I've had to ring the Garda [police] as a kind of concerned member of the Community because I couldn't get hold of a patient, you know?… In those cases, I felt my professional role did not get me anywhere. I was acting as a concerned citizen.” - P8.*

*“So probably done it as part of a chat or a conversation as supposed to think you know if Me as the pharmacist now in an advisory role or you know like so like I've never, I would have done it as part of a chat with the you know with the patient when they're there. But I wouldn't have seen it as the role of the pharmacist, if you know what I mean.” - P3.*

*“You can pick up on cues, but you see you're only going to be able to do that if you're out kind of talking to them, you know, because first of all, you need to build the relationship and then you're maintaining it.” – P7.*

*“But I see it as a community role that should be done just on a personal level.” - P5.*

Existing relationships with other community-based healthcare practitioners, particularly GPs were highlighted. Participants described situations where they intervened to support patients in collaboration with GP colleagues.

*“So, we would have phoned and talked to the GP and asked the GP were they noticing the same sort of thing or were they, you know. And just sort of said look, talk to the GP and sort out your concerns about with them. And then the GP got in touch with them, and then social support, social workers was brought in and there was a lot more support, saying put in place for that person as a result of it.” - P3.*

*“if I was worried about someone was, you know, the doctor or GP, I would phone their GP and say, you know, I'm a little bit worried about them. And I kind of, you know, would you, have you seen them recently or do you think you could talk to them that that's the other person?” - P1.*

*“But often I find what ends up happening would be that I would contact the GP to say You know that this person seems to be isolated, or maybe that they aren't accessing their like care properly or the medication properly through isolation.” – P4.*

Professional experience was identified as enabling pharmacists to identify patients experiencing loneliness through indirect means and engage with these patients.

*“I would not be actively looking for anything, but I suppose it is experience and intuition and knowing the person and we built up these relationships over the years that if they are off, you kind of know.” - P7.*

*“So you kind of have to be a bit more kind of alertness about finding them, you know, cause they're not obviously just gonna walk in the door and tell you.” - P2.*

*“I wouldn't feel one hundred percent confident because I feel like there's definitely elements that I might not consider yet just because like I am very newly qualified like I like I said, I'm quite young and like I might not have the same level of maturity to kind of identify things that someone who's maybe worked in the job for 20,25 years.”- P6.*

A deep level of rapport between patients and pharmacy staff teams was regarded as a valuable enabler for identifying and offering support to patients experiencing loneliness and social isolation. This relationship was described as currently providing a level of social as well as pharmaceutical care support to vulnerable patients.

*“You ensure that you either go out and do it yourself or you push a staff member who has a relationship with them, so not everyone talks to me. Not everyone wants to talk to, say my shop manager.” - P5.*

*“So naturally, in that setting, where I am, because all the staff know all the patient coming in and it's quite it's in a, you know or whatever and some of them are really good and they'll like, you know, they'll have the chats with them and they'll and, you know, they'll ask how they are and the staff are genuinely caring people.” —P3.*

*“I've one staff member in particular who will be even more proactive than I am. You know, she's like, just really caring, empathetic person, you know, and we go above and beyond…. Speaking to customer, you know they can recognize that.” – P8.*

*“And I do think, you know, maybe the pharmacist can be very busy and that's terrible. But you know, in some pharmacies I have worked in some of the OTC staff are excellent. They know everyone's circumstance.”- P9.*

*“We are very happy when they come in to chat to them and to do anything that we can and to deliver and to just maybe make that 10 minutes when they pop into the pharmacy in the week and a little bit of a social interaction.” —P1.*

*“You might be the only one that actually listens, and I find even just like you might just go out and think you are telling someone there is penicillin in the antibiotic and then you end up hearing their whole life story because they do not really have anyone else to listen to that.” – P6.*

### 3.6 Theme 4 - barriers to supporting patients experiencing SIL

Participants discussed several barriers to them effectively identifying and supporting patients experiencing loneliness or social isolation. A primary concern was a awareness and availability of resources that patients can be signposted or referred to. A lower level of awareness of meaningful supports for patients underpinned a hesitancy to discuss or raise issues related to loneliness.

“*All I can do is while they're in our pharmacy or the services we can give. That's all I can do is help with that moment, I really don't have a lot of access to anyone else, so it's, you know, it's not in my remit to do anything else at the moment, cause you know, during COVID, we did have a few phone numbers, but not anymore. It's not easy to get another person on board*”*– P1.*

*“It is hard for us to link in because I feel like we do not have the signposts.” - P7.*

*“You might not always know the public health nurse, or sometimes you would, and you know they are busy too.” – P9.*

*“I think I'd be OK picking it up, I think and, but I suppose the I suppose the thing I might probably find difficult is like, you know, OK, if there is an issue you agree that there is a bit of an issue with loneliness or social isolation is like what kind of you know what next?” - P2.*

A desire was expressed for enhanced training for pharmacists to support patients experiencing loneliness and social isolation. Participants had not received specific training in terms of supporting patients experiencing SIL.

*“I would say if pharmacists had more training, or not even more training, but it is they are more aware of the other supports, there are for people, then they would not have an issue contacting them.” - P3.*

*“I think if there was certain training in place, you could eventually get to the point that it is specific to identifying like loneliness and isolation and just having awareness of what particular verbal cues or like physical cues you might see.” - P6.*

*“But I do think like some of the training in college… we in fifth year had a lot of people coming in from like addiction centers and like mental health facilities where they'd be like, yeah, you might have heard of it like this.” – P4.*

*“I'm not sure how well trained we are in terms of like where to [refer patients experiencing SIL].” – P9.*

Participants also suggested that the workload pressures of community pharmacy practice prohibited them in engaging with patients who are experiencing issues such as social isolation to the extent that they felt necessary. Resource constraints including staffing were perceived to underpin this.

*“But in the midst of a busy day and you just don't have the capacity to do anything more, you know, and even quite often for the older person that's wanting to chat and because they're socially isolated, you're cutting it very short because it's busy now.” —P3.*

*“…unfortunately I'm looking for more and more out of my staff. There's no money, you know no one pays you to do any of this. You know, anything we do is just on our own back and there's nothing… And what I find that our pharmacy is just it's busier, there's less money in the dispensing and all that sort of stuff. And so it's just not feasible to employ someone for that. - P1.*

*“it's getting harder because pharmacies are getting busier. Umm, you know we are under a lot of pressure… not everywhere will have the time or the effort to put in to talk to these people”. – P8.*

*“Maybe sometimes it's a time factor that you're under pressure and the phone is ringing and this person still talking, and you know there are other people waiting for you and you are trying to wrap it up.” - P9.*

### 3.7 Survey study

95 regularly practicing community pharmacists consented to participate in the survey (response rate 95/6254, response rate 1.52 %). Respondents were predominantly experienced pharmacists with greater than 20 years' experience (*n* = 53, 55.8 %), with other participants less commonly represented (0–5 years *n* = 9, 9.5 %, 6–10 years *n* = 12, 12.6 %; 11–20 years *n* = 21, 22.1 %). A mix of community pharmacy roles were represented and a participants worked across a range of geographical settings (Rural location/village *n* = 14, 14.7 %; small town *n* = 27, 28.4 %; large town *n* = 28, 29.5 %; city suburb *n* = 22, 23.2 %, inner city *n* = 4, 4.2 %). The demographic makeup of survey respondents are broadly reflective of a recently reported survey of the register by the PSI.^24^

The majority of respondents reported encountering patients that they suspected to be experiencing SIL on a daily basis (*n* = 38, 40 %, 95 % CI: 30–50 %) or multiple times per week (*n* = 40, 42 %, 95 % CI:32 %–53 %). Respondents overwhelmingly reported encountering elderly patients experiencing SIL in the previous year (*n* = 91, 95.8 %, 95 % CI: 89 %–99 %), participants furthermore reported encountering individuals in the course of their professional practice experiencing SIL in the last year who were experiencing mental illness (*n* = 52, 54.7 %, 95 % CI:44–65 %), with long term physical health problems (*n* = 37, 39 %, 95 % CI: 29–50 %), recovering from or experiencing addiction (*n* = 33, 34.7 %, 95 % CI:25–45 %), with disabilities (*n* = 32, 33.7 %, 95 % CI:25–44 %), neurodiverse people (*n* = 24, 25.2 %, 95 % CI:17–35 %), members of migrant populations (*n* = 20, 21.1 %, 95 % CI: 14–30 %), young adults (*n* = 18, 18.9 %, 95 % CI: 12–29 %), individuals who had been widowed or bereaved (reported as “other”) (*n* = 6, 6.3 %, 95 % CI: 2–14 %), and LGBTI+ people (n = 6, 6.3 %, 95 % CI: 2–14 %).

Participants reported experiencing presentations of suspected SIL in practice manifesting as patients regularly presenting in the pharmacy without healthcare need, or presenting regularly with minor healthcare need (*n* = 78, 82 % 95 % CI:73–89 %), Other members of the pharmacy team (such as OTC staff or pharmacy technicians) identifying an individual as being at risk for experiencing loneliness or social isolation due to knowledge of their circumstances (*n* = 59, 62 %, 95 % CI: 52–72 %), individuals directly discussing feelings of loneliness (*n* = 57, 60 %, 95 % CI: 49–70 %), patients having difficulties with medication adherence exacerbated by loneliness (*n* = 53, 56 %, 95 % CI: 45–66 %) and patients having severe mental, physical, or social problems arising due to social isolation (*n* = 39, 41 %, 95 % CI: 31–52 %). Eight (*n* = 8, 8 %, 95 % CI: 4–16 %) participants selected the “other” option, with open ended responses largely referring to extended conversations of a non-pharmaceutical care related nature. One participant (*n* = 1, 1 %, 95 % CI: 0–7 %), answering other, reported never having identified a patient experiencing SIL.

Approaches to identifying patients who may be at risk of SIL reported were through discussion of wellbeing issues with patients or through observation of patient behaviour (*n* = 63, 66 %, 95 % CI 56–75 %), acting as a social contact/support for patients who may potentially be experiencing SIL (*n* = 52, 55 %, 95 % CI: 44–65 %), making other staff aware of patients who may be experiencing SIL or may be at risk of experiencing loneliness/social isolation (*n* = 59, 62 %, 95 % CI: 51–72 %) and building strong relationships with patients, making them more likely to feel comfortable discussing topics such as feeling lonely or isolated (*n* = 67, 70 %, 95 % CI 60–79 %).

A majority of participants either agreed (*n* = 33, 35 %, 95 % CI: 25–45 %) or strongly agreed (*n* = 27, 28 %, 95 % CI: 20–39 %) with the statement “supporting patients experiencing loneliness or social isolation is part of my role as a community pharmacist”. Twenty-three participants neither agreed nor disagreed with this statement, with smaller numbers disagreeing (*n* = 11, 12 %, 95 % CI: 6–20 %) or strongly disagreeing (n = 1, 1 %, 95 % CI: 0–7 %).). A large majority of participants (*n* = 79, 83 %, 95 % CI: 74–90 %) reported that their non-pharmacist colleagues play a role in identifying and supporting patients experiencing SIL. A majority of participants (*n* = 49, 52 %, 95 % CI: 41–62 %) reported agreement with the statement “I am comfortable in my ability to recognise and identify loneliness and social isolation in the pharmacy setting”, with smaller numbers strongly agreeing (*n* = 19, 20 %, 95 % CI: 13–30 %), neither agreeing or disagreeing (*n* = 22,23 %, 95 % CI: 15–33 %), disagreeing (n = 4, 4 %, 95 % CI: 1–11 %), strongly disagreeing (n = 1, 1 %, 95 % CI: 0–7 %).

A vast majority of participants (*n* = 78, 82 %, 95 % CI: 73–89 %) believed that barriers to addressing SIL in community pharmacy were present. These participants identified barriers related to the community pharmacy environment (financial pressures *n* = 44(46 %, 95 % CI: 36–57 %); time constraints *n* = 76 (80 %, 95 % CI: 70–87 %); staffing constraints *n* = 64 (67 %, 95 % CI: 57–77 %)), as well as barriers related to challenges in addressing SIL specifically (uncertainty about how to raise a conversation (*n* = 30, 32 %, 95 % CI: 22–43 %), uncertainty regarding availability of supports for patients (*n* = 38, 40 %, 95 % CI: 30–51 %), concerns regarding the stigmatised nature of loneliness (*n* = 24, 25 %, 95 % CI: 17–35 %). Forty-eight respondents (*n* = 48, 50 %, 95 % CI: 40–61 %) reported referring patients experiencing SIL to community-based supports in the past, describing referring patients to local community resources with Men's sheds the most commonly described referral service (*n* = 26) in open text responses.

## 4 Discussion

The data presented in this study strongly suggests that supporting lonely and socially isolated patients is an integral aspect of community pharmacy practice in Ireland with a majority of pharmacists reporting encounters with patients experiencing SIL, on a daily to almost daily basis. These findings highlight the potential of community pharmacy teams to contribute meaningfully to the management of SIL as a public health concern. A notable finding is the strong professional commitment expressed by pharmacists towards supporting patients experiencing SIL. This stands in contrast to a recent study of UK GPs who frequently considered addressing SIL as not part of their core professional roles and responsibilities.^11^ Indeed, this finding aligns with UK data suggesting an enthusiasm for pharmacy teams for engagement with social prescribing initiatives^25^ suggesting a unique professional orientation that may be leveraged to enhance support for patients experiencing SIL.

Pharmacists in this study consistently described SIL as a salient feature of their practice, often identified through indirect cues such as frequent non-healthcare visits or the emergence of health and wellbeing problems related to SIL. This aligns with existing data that suggests increased levels of loneliness among community pharmacy service users in Ireland,^13^ as well as high levels of SIL among the general population of Ireland.^9^^,^^26^

Elderly patients emerged as a key group of patients that pharmacists perceived as being both vulnerable to SIL and regular pharmacy service users. This finding is in line with expectations from the literature in terms of the disproportionate burden of loneliness in older age groups,^27^^,^^28^ as well as being more frequent users of pharmacy services.^13^ Pharmacists also reported encountering individuals experiencing SIL from further diverse groups with particular vulnerability to SIL including LGBTQ+ people,^29^ migrants,^30^ younger adults,^27^ people with mental health conditions^31^ including addiction^32^ disability^33^ and people with neurodiversity.^34^ This diversity of presentation suggests that person centered, tailored and flexible interventions are required to support lonely patients in community pharmacy settings. Co-design of pharmacy services in the context of these populations may represent a pathway towards impactful, tailored interventions.^35^

Pharmacists identified several enablers to supporting patients experiencing SIL in the pharmacy setting, including long term patient relationships, community embedded pharmacy staff and existing collaborations with other primary cares services such as general practitioners. Pharmacy teams represent a source of social support to potentially vulnerable patients, and already facilitate informal screening and referral of patients experiencing SIL. Indeed, the long-term community based relationships described in this study may facilitate the group membership and belonging that have been suggested to underpin the efficacy of existing SIL interventions.^36^

Significant barriers to effectively supporting patients experiencing SIL were also reported including unfamiliarity with existing community resources, time constraints and lack of training. Integration of pharmacy services with existing social care and support systems, particularly through formalised referral pathways, may support the delivery of SIL services in pharmacies. These findings furthermore support previous literature describing workload pressures and resource limitations in Irish community pharmacy.^37^

Taken together these findings suggest that without systemic support, the capacity of pharmacies to support patients experiencing SIL will remain constrained. McHugh-Power and Swader have argued that to effectively address loneliness as a public health issue, social connectivity must broadly be prioritised across policy domains at the national level.^10^ Accordingly, the promotion of social connectivity should be a central consideration in national health policy and in pharmacy service development. The role of pharmacies as a health and wellbeing hub is explicitly recognised in the most recent community pharmacy contract in Ireland^38^ however allocation of financial and staff resources to support meaningful engagement with patients experiencing SIL is not currently provided. This study provides evidence to support provision of these resources as an element of national health policy.

Irish national policy centres on social prescribing as an approach for the management of loneliness^10^ however, a broad range of further interventions have been demonstrated as effective in supporting patients experiencing SIL, particularly including group and individual based CBT approaches.^5^^,^^6^ Emerging evidence based public health guidance regarding social such as the Canadian public health guidelines for social connectivity^39^ and development of clinical guidelines^40^ may offer frameworks to guide a structured approach to SIL management in the pharmacy setting.

Current screening instruments commonly used in care settings to screen for loneliness^41^, ^42^, ^43^, 44. may not be contextually appropriate for use by community pharmacy teams given the preference for indirect communication surrounding loneliness expressed by participants in this study. Evaluation and adaptation of these tools in pharmacy settings or potentially development of bespoke validated screening tools may be a useful avenue for future research.

This study furthermore underscores the need for targeted educational support for pharmacists in understanding, recognising and managing SIL in the course of their practice, a theme consistently identified in the literature.^15^^,^^25^ This potentially includes development of training resources for both undergraduate and professional development settings. At an undergraduate level, social isolation is not currently broadly taught to prospective pharmacists and indeed is often absent from specialist undergraduate lifestyle medicine programmes.^45^ Undergraduate modules covering mental health, lifestyle medicine or patient counselling skills may be appropriate locations for the inclusion of evidence-based education on SIL. Peer-led learning approaches^46^ may be a mechanism to incorporate existing experience and skills into professional development training resources, while lived experience-informed and patient and public involvement (PPI) engaged training^47^ may enhance relevance and uptake of training resources in both settings.

### 4.1 Strengths and limitations

The current study presents a highly credible, triangulated data set generated via a mixed methods approach, with a strong basis in practical and lived experience of the context in question. The integration of qualitative and quantitative data enhances the robustness of the findings and provides a nuanced understanding of how SIL is encountered and managed in practice. It offers a methodological template for researchers in other jurisdictions to explore the presentation of SIL in their own pharmacy service context.

Several limitations must however be borne in mind when interpreting this data. Firstly, the data is firstly based in self-report where recall and social desirability biases may have influenced responses. Further to this response bias, where individuals with particular interests in SIL may have participated in this survey, may also be present and potentially confound interpretation of results. The recruitment strategy for the semi-structured interview component of the research was conducted to balance considerations of feasibility, inclusion of appropriate expertise and breadth of experience. However, this may introduce potential biases such as over representation of pharmacists with an existing interest in SIL, and underrepresentation of those not perceiving SIL as a salient component of practice.

Further, it is notable that the majority of respondents in both studies were more experienced pharmacists, which may have shaped the findings. As such the specific thoughts and experiences of early career pharmacists cannot be fully determined from this study. It is possible that more experienced pharmacists have developed a more refined perception of SIL in the course of their clinical practice, that is not yet present in less experienced colleagues, contributing to a potential response bias effect. It should however be noted that proportions reported in terms of experience are reflective of pharmacist ages and work locations as reported by a recent PSI survey of the register of pharmacists.^24^ Nonetheless, targeted research exploring the perceptions of early career pharmacists and pharmacy students may serve to illuminate further the themes described in this study.

Conceptual clarity may also be a limitation of this study. Participants in the qualitative interview component of this study appeared to perceive SIL from a perspective centred on the functional and structural aspects of social connectivity, more closely aligned with social isolation, and did not describe SIL in the emotional wellbeing terms that might align with a concept of loneliness. Although this presents a useful insight for the design and development of SIL training materials aimed at community pharmacists, it is possible that this lack of conceptual clarity may be a confounder for the interpretation of results, with participants reporting experience of social isolation or broader social determinant of health issues, rather than loneliness per se.

Although adequate for an exploratory study designed to triangulate qualitative research findings, a response rate of 1.52 % limits the broad generalisability of the survey results. This response rate may be a function of well characterised engagement and workload challenges in the community pharmacy setting,^37^ or may suggest small self-selecting population of pharmacists with an interest in SIL. This population may represent an engaged population who may be more likely to participate in future interventions or care delivery. This potential bias may be reinforced by the voluntary and non-incentivised nature of participation in this survey.

Further research, adopting observational approaches and interviews with wider groups stakeholders, such as patients and non-pharmacist team members is required to fully elucidate the precise nature of the presentation of SIL in the community pharmacy setting and develop acceptable, impactful evidence-based interventions.

## CRediT authorship contribution statement

**Jung-Ah Hong:** Writing – review & editing, Formal analysis. **Hebah Al-Rashed:** Writing – review & editing, Formal analysis. **Laura Rice:** Writing – review & editing, Formal analysis, Data curation. **Fabian F. Sweeney:** Writing – review & editing, Writing – original draft, Supervision, Methodology, Investigation, Formal analysis, Data curation, Conceptualization.

## Funding

This research did not receive any specific grant from funding agencies in the public, commercial, or not-for-profit sectors.

## Declaration of competing interest

The authors have no interests to declare.

## Appendix A Supplementary data

Supplementary material 1 Image 1

Supplementary material 2 Image 2
