# Supplementary material for: Pharmacist perceptions on the presentation of social isolation and loneliness (SIL) in community pharmacy settings in Ireland – A mixed methods study
Source: Explor Res Clin Soc Pharm. 2025 Nov 20;21:100686. doi: 10.1016/j.rcsop.2025.100686 (PMC12702109; doi:10.1016/j.rcsop.2025.100686)
Supplement: Supplementary file 1 — Supplementary material 1 [file mmc1.pdf]

Social Isolation in community pharmacy – semi-structured interview topic guide

Interview Introduction

My name is Dr Fabian Sweeney. I am a lecturer in the school of Pharmacy and Biomolecular sciences in RCSI

Confirm that participant has read PIL and that signed consent form has been received

The purpose of this interview is to gather your thoughts and experiences with regard to patients/customers/clients who may be experiencing loneliness or social isolation in the context of your professional practice

I will start by gathering some information about your overall professional experience and the nature of your current practice, then move on to discussing your experiences and thoughts regarding loneliness and social isolation.

The interview will take approximately 30 minutes

This interview will be recorded as outlined in the patient information leaflet but this interview is confidential and will be anonymised for analysis.

Do you have any questions before we begin?

Demographic and background details of participating pharmacists

- How many years have you been practicing as a pharmacist?
- How much of those have been as in community settings?
- ***Are you an owner of a pharmacy or an employee pharmacist?***
- Are you a supervising pharmacist, a support pharmacist or a locum pharmacist

Tell me about the pharmacy(s) that you work in.

Follow up questions

- Where is your pharmacy located
- Is your pharmacy independent or part a small (less than five pharmacies) or large chain (more than five pharmacies)?
- How many staff members work with you?

Research Question	Interview Questions
What are the attitudes of pharmacists towards social isolation and loneliness, and their perceived professional role in addressing this?	1. What do you understand by the terms loneliness and social isolation?<br>• What is your understanding of loneliness and social isolation in terms of it's impact on health and wellbeing?

	• How does this normally present in patients attending your pharmacy?<br>• Would you consider identifying and addressing social isolation to be a part of your professional role as a pharmacist?
What are the nature of interactions that take place in community pharmacy settings between community pharmacy staff and individuals potentially experiencing social isolation or loneliness?	2. What does loneliness and social isolation look like when it presents in your pharmacy?<br><br>3. **What kind of people. Are there more vulnerable, older people, younger people, mental illness? Why do you think this is?**<br><br>4. Are other team members in involved in supporting you identifying and supporting patients experiencing loneliness and social isolation?<br><br>*Possible ways to explore If a specific situation (if mentioned?)*<br>• *What happened?*<br>• *Were any other team members involved in this situation?*<br>• *How did you determine that this person was experiencing loneliness/social isolation?*<br>• *What did you do? How did you support this patient?*<br>• *How do you feel the situation went?*<br>• *Does this represent a typical situation in your practice?*<br>• ***What did you feel was your professional role is in this situation?***<br>• *Can you tell me about any other times where you encountered social isolation or loneliness in the course of your professional practice?*
What is the level of perceived self-efficacy among pharmacists in identifying and supporting patients experiencing social isolation and loneliness?	5. How comfortable are you in your ability to raise to topic of connectedness or loneliness with a patient? Why do you say this?<br><br>6. How comfortable are you in your capability to determine if a patient is experiencing social isolation or loneliness? Why do you say this?

	7. How comfortable would you be in your capability to support a patient who is experiencing social isolation or loneliness? Why do you say this? What might this look like?<br><br>8. What kinds of support do you think would be useful to you to increase your ability to identify and address social isolation and loneliness as part of your professional role?<br><br>*Previous research has suggested identifying and supporting patients at risk of loneliness using models based around identifying patients*<br>• *Structured questionaires delivered by a pharmacy team (just loneliness/broader social issues)*<br>• *Direct liaison with partners in the community/social prescribing co-ordinators/ state-bodies*<br>• *Training staff members to specifically work with patients on social determinants of health issues.*
	9. Is there anything else you would like to add?
