# Supplementary material for: Pharmacist perceptions on the presentation of social isolation and loneliness (SIL) in community pharmacy settings in Ireland – A mixed methods study
Source: Explor Res Clin Soc Pharm. 2025 Nov 20;21:100686. doi: 10.1016/j.rcsop.2025.100686 (PMC12702109; doi:10.1016/j.rcsop.2025.100686)
Supplement: Supplementary file 2 — Supplementary material 2 [file mmc2.pdf]

Loneliness & Social Isolation Questionnaire

Please **do not** disclose any identifiable details about yourself, patients, or colleagues when completing this survey.

* Required

1. I have read and understood the Information Leaflet about this research project. The information has been fully explained to me and I have been able to contact the researchers to ask questions if applicable, all of which have been answered to my satisfaction. *

   ◯ Yes

   ◯ No - by selecting this option you will exit the survey

2. I understand that I do not have to take part in this study and can choose not to complete the survey. If I choose to proceed, I understand that my survey responses will be pooled with those of other participants in an unidentifiable manner. Therefore, it will not be possible to withdraw my answers once I have submitted my online response. *

   ◯ Yes

   ◯ No - by selecting this option you will exit the survey

3. I am aware of the potential risks, benefits and alternatives of this research study. *

   ◯ Yes

   ◯ No - by selecting this option you will exit the survey

4. I have been given a copy of the information leaflet by email for my records. *

   ◯ Yes

   ◯ No - by selecting this option you will exit the survey.

5. I consent to take part in this research study having been fully informed of the purpose, risks, benefits and alternatives. *

   ◯ Yes

   ◯ No - by selecting this option you will exit the survey.

6. I give informed explicit consent to have my data processed as part of this research study. *

- Yes
- No - by selecting this option you will exit the survey.

7. Do you regularly practice as a pharmacist in a community setting in Ireland? *

- Yes
- No - by selecting this option you will exit the survey.

8. How many years have you been practicing as a community pharmacist? *

- 0-5 years
- 6-10 years
- 11-20 years
- 20+ years

9. Please select which best describes your role: *

- Superintendent pharmacist
- Supervising pharmacist
- Support pharmacist
- Locum pharmacist

10. What best describes the setting you work in? *

- Rural location/village
- Small town
- Large town
- City suburb
- Inner city

11. How often do you encounter patients who you suspect may be experiencing loneliness or social isolation in your normal practice? Select the option which closest fits your opinion. *

- ◯ Daily
- ◯ Several times per week
- ◯ Once per week
- ◯ Once per month
- ◯ Less than once per month
- ◯ Never

12. How often do you encounter patients who have explicitly mentioned that they are experiencing loneliness or social isolation in your normal practice? Select the option which closest fits your opinion. *

- ◯ Daily
- ◯ Several times per week
- ◯ Once per week
- ◯ Once per month
- ◯ Less than once per month
- ◯ Never

13. Evidence suggests that certain populations are more susceptible to experiencing loneliness or social isolation. In the past year, have you encountered patients with suspected loneliness or social isolation who are members of any of the following groups in the course of your practice? Please select all that apply. *

- ☐ Older people
- ☐ Young adults
- ☐ People with disabilities
- ☐ Neurodiverse people
- ☐ People experiencing mental illness
- ☐ People experiencing or recovering from addiction
- ☐ People with one or more long-term physical health conditions
- ☐ People from migrant populations
- ☐ LGBTI+ people
- ☐ Other:

14. If you selected "other" as part of the previous question, please explain your response below.

15. Which, if any, of the following situations have suggested to you that a patient is potentially experiencing loneliness or social isolation? Please select all that apply. *

- ☐ Patients regularly presenting in the pharmacy without healthcare need, or presenting regularly with minor healthcare needs
- ☐ Patients having difficulties with medication adherence, exacerbated by social isolation
- ☐ Patients having severe mental, physical, or social problems arising due to social isolation
- ☐ Individuals directly discussing feelings of loneliness
- ☐ Other members of the pharmacy team (such as OTC staff or pharmacy technicians) identifying an individual as being at risk for experiencing loneliness or social isolation due to knowledge of their circumstances
- ☐ Other:
- ☐ I have never identified any patients as potentially experiencing loneliness or social isolation

16. If you selected "other" as part of the previous question, please explain your response below.

17. How much do you agree or disagree with the following statement: "supporting patients experiencing loneliness or social isolation is part of my role as a community pharmacist"? *

- ◯ Strongly agree
- ◯ Agree
- ◯ Neither disagree nor agree
- ◯ Disagree
- ◯ Strongly disagree

18. Please explain your response to the previous question. *

19. Preliminary research has indicated that other members of the pharmacy team, such as OTC staff or pharmacy technicians, play a role in identifying and supporting loneliness and social isolation in the pharmacy setting.

    Is this something that other members of your pharmacy team are involved in? *

    - ○ Yes
    - ○ No

20. If you answered "yes" to the previous question, please select below which behaviours your pharmacy team members are involved in. Please select all that apply.

    - ☐ Identifying patients who may be at risk of loneliness or social isolation, for example, through discussion of wellbeing issues with patients or through observation of patient behaviour
    - ☐ Acting as a social contact/support for patients who may potentially be experiencing loneliness or social isolation
    - ☐ Making other staff aware of patients who may be experiencing loneliness/social isolation or may be at risk of experiencing loneliness/social isolation
    - ☐ Building strong relationships with patients, making them more likely to feel comfortable discussing topics such as feeling lonely or isolated
    - ☐ Other

21. If you selected "other" as part of the previous question, please explain your response below.

22. How much do you agree or disagree with the following statement: “I am comfortable in my ability to recognise and identify loneliness and social isolation in the pharmacy setting”. *

    - ○ Strongly agree
    - ○ Agree
    - ○ Neither disagree nor agree
    - ○ Disagree
    - ○ Strongly disagree

23. Please explain your response to the previous question. *

24. How might you approach raising the topic of loneliness or social isolation with a patient who you suspect might be experiencing loneliness or social isolation? *

25. Do you feel there are barriers to you identifying or addressing loneliness or social isolation in the community pharmacy setting? *

- ◯ Yes
- ◯ No

26. If you answered "yes" to the previous question, please select all barriers that you think are present.

- ☐ Financial pressures of community pharmacy environment
- ☐ Time constraints
- ☐ Staffing constraints
- ☐ Uncertainty around how to raise a conversation about loneliness
- ☐ Uncertainty about supports which may be available to people experiencing loneliness
- ☐ Concerns surrounding loneliness as a stigmatised topic
- ☐ Other

27. If you selected "other" as part of the previous question, please explain your response below.

28. There is evidence that referring isolated patients to non-clinical community supports, i.e. “social prescribing”, can have significant benefits for their overall health and wellbeing. Some such supports include Men’s Sheds, Park Runs, Social Prescribing Ireland Services, and other local community organisations.

Have you ever referred patients to any of these services? If yes, please briefly describe the nature of these referrals in the next question. *

- ◯ Yes
- ◯ No

29. Please briefly describe the nature of any referrals you have made to social prescribing services.

30. What do you feel might support you in identifying, addressing and supporting those experiencing loneliness in the community setting? *

31. Are there any other thoughts on this topic you would like to share at this time?

This content is neither created nor endorsed by Microsoft. The data you submit will be sent to the form owner.

Microsoft Forms
